# The Genetic Landscape of Paediatric Cataract in Saudi Arabia: A Two-Decade Cohort with Novel Variants, Genotype–Phenotype Correlations, and Bioinformatic Analysis

**DOI:** 10.3390/jcm15062420

**Published:** 2026-03-21

**Authors:** Mashael Alsugair, Fay Alsuhaym, Hitham Aldharee, Saif Alobaisi, Saeed Alsharani, Saud Alwatban, Muhannad A. Alnahdi, Mohammed Al Balwi

**Affiliations:** 1 College of Medicine, Qassim University, Buraidah 51452, Saudi Arabia; 2 College of Medicine, Princess Nourah bint Abdulrahman University, Riyadh 11564, Saudi Arabia; 3 Department of Pathology, College of Medicine, Qassim University, Buraidah 51452, Saudi Arabia; 4 King Abdullah Specialised Children’s Hospital, National Guard, Riyadh 11481, Saudi Arabia; 5 Department of Ophthalmology, National Guard Hospital, Riyadh 11426, Saudi Arabia; 6 Boston Consulting Group, Singapore 068897, Singapore; 7 Department of Ophthalmology and Vision Sciences, Temerty Faculty of Medicine, University of Toronto, Toronto, ON M5S 1A1, Canada; 8 Department of Pathology and Laboratory Medicine, King Abdulaziz Medical City, Ministry of National Guard Health Affairs, Riyadh 11426, Saudi Arabia

**Keywords:** paediatric cataract, genetics, syndromic cataract, cataract genesis, Saudi Arabia

## Abstract

**Background/Objectives:** Paediatric cataract is among the most common treatable causes of childhood blindness, caused by a genetically diverse disorder with variable clinical features. Although genetic factors significantly contribute to the development of paediatric cataracts, recent data on their genetic makeup and genotype–phenotype relationships in Saudi Arabia is limited. This study aims to investigate the genetic spectrum, inheritance patterns, and genotype–phenotype correlations of paediatric cataract in a Saudi population over twenty years. **Methods:** We conducted a retrospective cohort study of children diagnosed with congenital or juvenile cataracts between 2000 and 2019 at two major referral centres in Riyadh. Clinical, ocular, and systemic data were collected through multidisciplinary evaluations. Genetic analysis involved whole-exome and whole-genome sequencing performed at College of American Pathologists (CAP)-accredited laboratories. Variant interpretation was supported by bioinformatic and Artificial Intelligence (AI) prediction tools. Genotype–phenotype relationships were systematically analysed. **Results:** The study included 28 cases of genetically confirmed paediatric cataracts. Variants classified as pathogenic or likely pathogenic were identified in 13 genes. Autosomal recessive inheritance was predominant, with many patients exhibiting homozygous variants, often due to consanguinity. Two novel variants were identified in the Collagen Type XVIII Alpha 1 Chain (*COL18A1*) and the RAB3 GTPase-activating protein catalytic subunit 2 (*RAB3GAP2*) genes. Considerable phenotypic variability was observed, even among patients with the same mutation, particularly those with the recurrent CRYBB1 c.171del (p.Asn58fs) mutation. Syndromic cataracts were more frequently associated with loss-of-function variants and multisystem features. **Conclusions:** This study offers updated insights into the genetics and clinical presentation of paediatric cataract in Saudi Arabia. It highlights high genetic diversity, unique inheritance patterns, and notable genotype–phenotype variability, emphasising the importance of early genetic testing and multidisciplinary assessment for improved diagnosis, management, and counselling.

## 1. Background

A cataract is a lenticular opacity that prevents light from reaching the retina and is the most prevalent treatable cause of childhood blindness. Although typically associated with age-related degeneration, the condition may also occur in paediatric populations [1]. Globally, paediatric cataract affects approximately 0.01% of children, with a median prevalence of 1.03 per 10,000, totalling up to 200,000 cases worldwide. The prevalence rate is 1.69 per 10,000 children, corresponding to roughly 314,000 new cases annually [2].

Paediatric cataract is clinically classified as unilateral, bilateral, congenital, or acquired. Its aetiology may be genetic, encompassing hereditary and disease-associated cataracts, or attributable to trauma, drug-induced causes, maternal infections, or iatrogenic factors [1,3]. Certain childhood cataracts remain idiopathic, diagnosed only after all known causes have been ruled out. Some cases present with leukocoria (a white reflex in the pupil), others with difficulty tracking nearby objects or recognising familiar faces, some with microphthalmos (abnormally small eyes), others with buphthalmos (enlarged eyes), and some with nystagmus (rapid eye movements). The only effective intervention for cataracts that substantially impair vision is prompt surgical removal, which is essential to preserve the optic nerve and prevent permanent vision loss [1].

The genetic aetiology of paediatric cataract may manifest as either an isolated lenticular opacity, such as microcornea (minimal ophthalmic involvement), or as a syndromic cataract associated with systemic organ dysfunction. It is estimated that 8–29% of childhood cataracts with genetic causes are reported in the developed world [4]. A genetic variant causing cataract does not invariably present in the initial years of life. Senile cataract, which occurs after age 45, may be linked to genetic susceptibility. A cataract present at birth is regarded as congenital, typically resulting from genetic anomalies or intrauterine trauma, whereas one that develops after the first year of life is classified as juvenile [5]. Scientific research has identified 41 genes implicated in the development of solitary infantile cataracts [6].

Previous studies have demonstrated considerable geographic variation in the genetic causes of paediatric cataracts. For example, investigations from North America and Europe often report a predominance of autosomal dominant variants, particularly in crystallin genes responsible for maintaining lens transparency [7,8,9]. In contrast, studies from Asian populations, including China, have identified a broader spectrum of variants affecting crystallin and other cataract genes [10]. These international findings highlight the importance of conducting population genetic studies to better understand the regional genetic architecture of paediatric cataracts. In the *MENA* region, numerous studies have documented associations between genetic variants and paediatric cataracts across various ethnic groups [11]. The principal inheritance pattern observed in this region is autosomal recessive. In 2012, a pioneering study from the Kingdom of Saudi Arabia, using molecular diagnostic testing for paediatric cataracts, found that genetic factors accounted for 79% of cases [12].

Recent advances in genomic technologies now enable the simultaneous analysis of a wide range of cataract-associated genes [13]. Despite this progress, there remains a lack of updated data on the epidemiology and genetic architecture of paediatric cataracts in Saudi Arabia. The most recent population-based study, published in 1989, reported a prevalence of 14.7 per 10,000 children aged 5 to 18 years, which is considerably higher than global reported estimates, highlighting the need for updated investigations.

In populations with high rates of genetic disorders and consanguinity, such as Saudi Arabia, the genetic spectrum and inheritance patterns may differ from those observed in Western populations. Therefore, this study aims to characterise the genetic variants, inheritance patterns, and genotype–phenotype correlations of paediatric cataracts in a Saudi cohort over twenty years, which could provide valuable insights for this region.

## 2. Materials and Methods

### 2.1. Study Setting and Design

The study was conducted within the Department of Ophthalmology at King Abdulaziz Medical City (KAMC), a tertiary healthcare facility in Riyadh, Saudi Arabia, from 2000 to 2015. Subsequently, the research was transferred to King Abdullah Specialized Children Hospital (KASCH) for the period 2016 to 2019. KASCH commenced operations in 2016 as the Kingdom’s first medical referral institution dedicated to paediatric care. The study was approved by the Institutional Review Board (IRB) of King Abdullah International Medical Research Centre (KAIMRC) (approval no. RC20/318/R). Due to the retrospective design and the use of anonymised clinical data, informed consent was not required.

The study presents a retrospective cohort analysis of paediatric patients referred to the ophthalmology department from 2000 to 2019. Cases categorised under the code H26.0—Infantile and juvenile cataract—in accordance with the International Classification of Diseases, 10th edition (ICD-10), were retrieved.

### 2.2. Cases, Inclusive and Exclusive Criteria

All paediatric cataract cases diagnosed over a 20-year period were reviewed. A cataract was classified as congenital if it presented during the first year of life and as juvenile if it developed after the first year but before 10 years of age. Patients were excluded if the cataract was attributable to metabolic disorders such as Fabry disease, galactosemia, or Wilson disease; intrauterine infections or infections associated with cytomegalovirus, rubella, syphilis, toxoplasmosis, or varicella; or if they had a history of trauma, diabetes mellitus, lenticular opacity due to neoplastic growth, persistent hyperplastic primary vitreous, or a toxic cataract related to corticosteroid use or radiation exposure. 246 patients were evaluated against the inclusion criteria. After excluding cases of secondary cataract and those with incomplete data, 28 patients were included in the study. Cases were classified as having a genetic origin solely if cataract-related variants were confirmed by genetic testing.

### 2.3. Clinical, Ophthalmic, and Genetic Assessments

A cataract is typically identified during newborn screening through the red reflex test performed by a primary care provider or paediatrician, or later if the child develops symptoms. At KAMC–KASCH, a multidisciplinary assessment was conducted for cataract cases, involving the divisions of ophthalmology, neonatology, general paediatrics, genetics, metabolism, and pathology. The genetic evaluation was guided by the child’s growth and development, systemic conditions, family history, and specific ophthalmic indicators that warranted additional genomic investigation.

Ocular examinations were conducted by expert ophthalmologists using a slit-lamp biomicroscope to classify the morphology of lenticular opacities and to identify associated ocular manifestations, such as microphthalmia, microcornea, and nystagmus. A dilated fundus examination was also performed to exclude other conditions that mimic an abnormal red reflex and to evaluate for associated retinal, glaucomatous, and optic nerve lesions. In certain cases, examinations under anaesthesia were carried out. Strabismus was assessed using the cover–uncover test. High myopia was defined as an objective spherical equivalent refractive error exceeding −6.00 diopters.

Developmental delay was characterised by a child’s inability to achieve standard developmental milestones in at least two of the following domains by the expected age: cognitive, social, language, fine motor, or gross motor skills. Gross motor development was assessed by evaluating muscle tone, muscle movement, and deep tendon reflexes. Craniofacial deformities, dysmorphic features, and skeletal abnormalities—including foot polydactyly, pes planus, talipes equinovarus, scoliosis, limb shortening, dwarfism, multiple joint dislocations, or multiple bone fractures—were examined clinically, with additional imaging studies performed as necessary.

Cardiac defects included atrial septal defect, ventricular septal defect, patent ductus arteriosus (PDA), and any cyanotic heart defect (e.g., coarctation of the aorta, transposition of the great arteries). All patients were routinely assessed for growth, and any regression or failure to achieve expected milestones was evaluated for possible failure to thrive.

Hearing loss was documented using at least one of the following objective measures, as clinically indicated: visual reinforcement audiometry, audiometry, evoked otoacoustic emissions, or auditory brainstem response. Genital anomalies included hypospadias, undescended testes, and micropenis. Anatomical upper airway obstruction was considered significant if distortion of the pretracheal passage caused moderate to severe respiratory distress (e.g., nasolabial deformity, mass, enlarged tonsils, enlarged tongue, or hypotonia of the pharyngeal muscles).

Predictors of parenchymal brain damage or deformity were identified through clinical and/or radiological assessments, including congenital microcephaly, cerebral malformations, generalised spasticity, or brain atrophy. A child was classified as having pancytopenia if there was a concomitant reduction in haemoglobin levels (<10 g/dL), white blood cell count (<4000), and platelet count (<150,000).

Genetic testing was performed on the probands. Testing, including Whole Genome Sequencing (WGS) and Whole Exome Sequencing (WES), was conducted at CAP-accredited commercial genomic laboratories. Inheritance patterns were assigned based on zygosity, known gene-specific inheritance, and family history, and are summarised in Supplementary Table S1.

### 2.4. Data Collection and Ethical Approval

The medical records were reviewed for relevant study variables. Files from 2000 to 2015 were collected as paper or scanned documents, while files from after 2016 were collected electronically via BESTCare v.2. Data confidentiality and anonymity were maintained throughout all phases of the study.

### 2.5. Bioinformatic Tools and Computational Prediction Methods

#### 2.5.1. Predicting Pathogenicity of Variants

Several bioinformatics tools were used to assess whether the detected variants were disease-causing. PHD-SNP [14] predicts whether a non-synonymous single-nucleotide polymorphism (nsSNP) is associated with disease or is neutral, using a support vector machine trained on annotated variant datasets. Variants predicted to be “disease-related” were considered potentially pathogenic. SNAP2 [15] applied a neural network to analyse the sequence and predict whether a variant was “damaging or “neutral.”

SNAP2 predictions were obtained using the SNAP2 server, which classifies amino acid substitutions as either effect (damaging) or neutral, and provides a prediction confidence value ranging from 0 to 1, indicating the strength of the prediction.

PANTHER [16] relied on evolutionary conservation, with interpretation based on the PSEP value: scores less than 200 were regarded as probably benign; scores less than 450 were considered possibly damaging; and scores greater than 450 were deemed likely damaging. SIFT [17] predicts whether an amino acid substitution affects protein function based on sequence homology and amino acid conservation, with scores ≤0.05 classified as deleterious and scores above 0.05 regarded as tolerated. Meta-iSNP [18] predicted the functional impact of amino acid substitutions; scores below −2.5 indicated potential damage, whereas higher scores were regarded as neutral. PolyPhen-2 [19] assessed the protein sequence and structural model to determine the impact of variants on protein stability. PolyPhen-2 probability scores range from 0 to 1: values near 0 suggest a benign effect, while scores close to 1 indicate a likely damaging impact on protein function.

#### 2.5.2. AlphaMissense Variants Pathogenicity Prediction

AlphaMissense [20], an artificial intelligence model trained on extensive human and primate datasets, was used to assess the impact of missense variants on protein function. The model generated a probability score from 0 to 1, with higher values indicating a greater likelihood of pathogenicity and lower values suggesting a benign effect. Variants with AlphaMissense scores ≥0.56 were considered likely pathogenic, while lower scores were regarded as likely benign.

#### 2.5.3. Predicting Protein Stability

The impact of amino acid substitutions on protein stability was assessed using MUpro [21] and I-Mutant 2.0 [22]. Both software tools use machine learning algorithms and produce a stability score; lower scores indicate reduced protein stability, compromised folding, decreased enzymatic activity, and increased susceptibility to rapid degradation.

#### 2.5.4. Three-Dimensional (3D) Structure Prediction

The 3D structures of the proteins and their variants, which were predicted to be pathogenic by all the bioinformatic tools, were modelled using the Iterative Threading ASSEmbly Refinement (I-TASSER) tool (https://seq2fun.dcmb.med.umich.edu//I-TASSER/, accessed on 12 March 2026). Additionally, the Missense3D tool (https://missense3d.bc.ic.ac.uk/missense3d/, accessed on 13 March 2026) was used to predict any potential structural damage in the 3D protein structures caused by amino acid substitutions. The UCSF Chimera software (https://www.cgl.ucsf.edu/chimera/, accessed on 13 March 2026) was also employed to visualise the 3D structures and overlay the normal and mutated proteins.

## 3. Results

The study includes 28 patients divided into three groups based on clinical features: those with only cataracts (pure cataract), those with cataracts plus ocular abnormalities but no syndromic features (non-syndromic), and those with cataracts along with systemic defects (syndromic). Genetic analysis identified 13 genes harbouring 17 variants associated with paediatric cataract, including 3 novel variants.

### 3.1. Patient Demographic and Presentation

The cases comprised both males and females, with males accounting for 57.14% and females for 42.86% Table 1. Most patients were diagnosed very early, typically during the neonatal period or within the first few months of life. Congenital cataract was the most common type, occurring in 82.13% of patients, while fewer patients were diagnosed with juvenile cataract between the ages of 2 and 6 years. Bilateral cataract was noted in nearly all cases, with only one patient showing unilateral involvement. Overall, the main clinical features were early age at presentation and bilateral involvement.

### 3.2. Spectrum of Identified Genetic Variants

Pathogenic (P) and likely pathogenic (LP) variants were identified in multiple genes associated with congenital and paediatric cataracts, including *CRYBB1*, *SIL*, *COL18A1*, *RAB3GAP1*, *RAB3GAP2*, *PEX7*, *EPHA2*, *AGK*, and *GNPAT* (Table 2). Variants of uncertain significance (VUS) were also found in *COL18A1*, *FYCO1*, *CRYBA4*, *MAF*, and *GRIA3*. Most cases showed autosomal recessive inheritance, with affected individuals often homozygous, particularly in families with reported consanguinity. Fewer cases involved autosomal dominant variants, and one patient carried an X-linked recessive variant. Several families reported a family history of cataracts.

Importantly, two novel variants were identified in this cohort: COL18A1 c.355delG p.(Val119fs) and RAB3GAP2 c.1348dup p.(Ser450Phefs*36) Table 2. These variants have not been previously reported in major population or disease variant databases, suggesting they are previously undescribed alterations linked to paediatric cataract and related phenotypes. Both variants are predicted to cause frameshift mutations, which generally lead to premature termination of translation and the production of truncated proteins that may disrupt normal protein function.

### 3.3. Genotype–Phenotype Correlation

A wide range of cataract types was observed, including nuclear, anterior polar, lamellar pulverulent, sutural, total, posterior polar, and anterior subcapsular cataracts Table 3. The most common phenotypes across various genetic backgrounds were nuclear and anterior polar cataracts. Some patients also had additional eye conditions such as strabismus, esotropia, exotropia, glaucoma, nystagmus, optic nerve atrophy, and microphthalmia. Non-syndromic cataracts without other eye issues (category 1) were rare, while those with additional ocular features (category 2) represented a significant portion of the group. Syndromic cataracts (category 3) occurred in multiple patients and were associated with pathogenic variants in genes such as *SIL*, *COL18A1*, *RAB3GAP2*, *PEX7*, *MAF*, *RAB3GAP1*, *GNPAT*, and *AGK*. These cases showed diverse systemic phenotypes, including developmental delay, hypotonia, microcephaly, craniofacial dysmorphisms, failure to thrive, congenital retinal dystrophy, cerebellar atrophy, and multisystem involvement, consistent with recognised syndromes such as Knobloch, Martsolf, Warburg microsyndrome, Ayme-Gripp, Senger, and rhizomelic chondrodysplasia punctata. Variants resulting in frameshift, nonsense, and intronic changes were more strongly associated with syndromic phenotypes, whereas missense variants were mostly observed in non-syndromic or ocular-limited cases.

The CRYBB1 c.171del (p.Asn58fs) variant was found in multiple unrelated patients and was associated with different types of cataracts, including anterior polar, nuclear, lamellar pulverulent, and sutural cataracts. Despite all carrying the same homozygous frameshift mutation, individuals show a range of eye conditions—from isolated cataracts to strabismus and glaucoma—highlighting phenotypic variability. The *COL18A1* c.355delG p.(Val119fs) frameshift mutation was linked to total cataract and complex eye features such as exotropia, high myopia, congenital retinal dystrophy, and spontaneous rotational nystagmus, as well as systemic signs of Knobloch syndrome like speech delay and intellectual disability. Likewise, the *RAB3GAP2* c.1348dup p.(Ser450Phefs*36) was identified in a syndromic case presenting with cataract, glaucoma, strabismus, and optic nerve atrophy, along with systemic signs such as microcephaly and features of Martsolf syndrome. Overall, these findings reveal considerable clinical diversity, even among patients with variants in the same gene.

### 3.4. Bioinformatic Analysis

#### 3.4.1. Assessing the Pathogenic Potential of Identified VUS Variants

The VUS variants listed in Table 2, including *CRYBA4* c.206T>C (L69P), *MAF* c.188C>G (P36A), *COL18A1* c.803C>T (A23V), and *GRIA3* c.2189G>C (G730E) were assessed using two groups of computational tools. The first group comprised traditional methods such as Meta-SNP, PolyPhen-2, PANTHER, and SIFT, while the second included AI and machine learning systems such as PHD-SNP and SNAP2. The combined results indicated that only some variants, especially *CRYBA4* c.206T>C (L69P) and *GRIA3* c.2189G>C (G730E), showed consistent pathogenicity predictions across the tools Table 4.

#### 3.4.2. AlphaMissense Variants Pathogenicity Prediction

The same variants were reevaluated using the AlphaMissense tool, which employs a deep learning model. As shown in Table 5, the *CRYBA4* c.206T>C (L69P) and *GRIA3* c.2189G>C (G730E) variants received very high probability scores (0.810 and 0.991, respectively), suggesting they are likely pathogenic. Despite using different algorithms and scoring methods, AlphaMissense’s results align with earlier identified damaging variants, providing evidence for their role in disease manifestation.

#### 3.4.3. Predicting Protein Stability

The L69P and G730E variants were assessed for their effects on protein stability using the I-Mutant and MUpro tools. As shown in Table 6, both methods predict decreased stability for each variant. I-Mutant indicated that *CRYBA4* c.206T>C (L69P) variant reduces stability, with a reliability index of 5. MUpro also forecasted a decline, with a score of −2.17, suggesting the protein’s stability is compromised and its folding is affected. Similarly, *GRIA3* c.2189G>C (G730E) variant shows comparable decreases (reliability index = 4 in I-Mutant and −1.103 in MUpro), implying the mutation may disrupt the protein backbone and impair function. These findings support earlier bioinformatics predictions, which indicated that these variants are not only deleterious in sequence-based models but also pose a threat to structural integrity.

#### 3.4.4. 3D Structure Prediction

**Figure 1:**
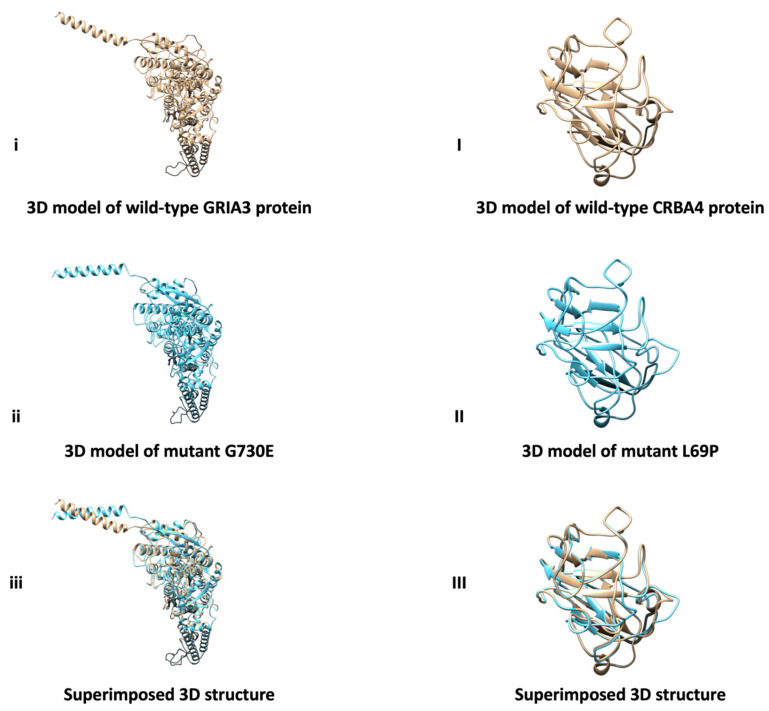
The three-dimensional (3D) analysis of the normal GRIA3 and CRBA4 proteins and the detected variants. (**i**,**ii**,**iii**) show the 3D models of the wild type GRIA3, the G730E variant, and the superimposed structure, respectively. (**I**,**II**,**III**) display the 3D models of the wild-type CRBA4, the L69P variant, and the superimposed structure, respectively.

TASSER, UCSF Chimera, and Missense3D tools were used together to evaluate the structural differences and effects between the normal GRIA3 and CRBA4 proteins and the detected variants (G730E and L69P). The results indicate that the variants do not cause clear structural damage to the normal proteins. Additionally, the 3D models of the mutant proteins were superimposed onto the wild-type model to assess structural similarity (Figure 1). The findings suggest that both variants are very similar to the corresponding wild-type protein.

## 4. Discussion

This study offers insight into the genetic and clinical spectrum of paediatric cataract in a Saudi cohort and highlights the significant genetic diversity underlying this condition. Consistent with previous reports from highly consanguineous populations, autosomal recessive inheritance was common in this cohort, with most patients carrying homozygous variants. Studies from the Middle East and Asia have similarly documented a high prevalence of recessive cataract genes in populations where consanguineous marriages are common [12,23,24,25]. Conversely, studies from North America and Europe generally report autosomal-dominant inheritance as the most common pattern in hereditary cataracts [4,13]. These differences underscore the importance of considering population genetic structures when interpreting cataract inheritance patterns and developing diagnostic strategies.

A notable finding in this cohort was the recurrent *CRYBB1* c.171del (p.Asn58fs) variant, observed in several unrelated patients. Alterations in *CRYBB1* are well-established causes of congenital cataract and are most inherited in an autosomal-dominant pattern [4]. However, in this cohort, the variant was homozygous, suggesting an autosomal recessive inheritance pattern. Similar recessive cases with the same variant have been reported in populations with high rates of consanguinity, indicating that inheritance patterns may depend on the mutation type and zygosity [12,25,26]. The frameshift nature of the c.171del mutation probably results in a complete loss of protein function, which might explain why disease expression in this group required biallelic variants. This highlights the complexity of the mechanisms underlying disease in crystallin genes and suggests that both dominant-negative and loss-of-function processes may contribute to cataract development, depending on the specific mutation.

Though the same *CRYBB1* c.171del (p.Asn58fs) variant was present in several patients, their phenotypes varied significantly. The types of cataracts ranged from lamellar and anterior polar opacities to nuclear and sutural opacities, with some patients also experiencing other ocular conditions, including strabismus and glaucoma. Similar phenotypic diversity has been observed in crystallin-related cataracts, where the same mutation can produce different clinical features even within the same family [11]. This variation likely results from the effects of modifier genes, environmental influences, or epigenetic factors, suggesting that genotype alone may not fully determine the clinical picture. The study also identified several genes linked to syndromic cataracts, including *COL18A1*, *RAB3GAP1*, *RAB3GAP2*, *PEX7*, *GNPAT*, and *AGK*, which are known to cause multisystem developmental disorders. Patients with variants in these genes often exhibit neurological, skeletal, or metabolic abnormalities alongside ocular disease, aligning with previously described syndromes such as Knobloch syndrome, Martsolf syndrome, Warburg micro syndrome, and rhizomelic chondrodysplasia punctata. These findings underscore the importance of recognising paediatric cataracts not only as a standalone ocular condition but also as a potential sign of broader systemic disease.

Interestingly, two previously unreported variants were identified in *COL18A1* p. (V119fs) and *RAB3GAP2* p. (Ser450Phefs*36), both predicted to cause frameshifts resulting in truncated proteins. Variants in *COL18A1* disrupt collagen XVIII, a structural component of basement membranes that plays a crucial role in ocular development and retinal integrity [27,28,29]. Similarly, *RAB3GAP2* encodes a component of the RAB3 GTPase-activating protein complex that plays a role in vesicle trafficking and neuronal development. Variants in this gene have been associated with Martsolf syndrome and other neurodevelopmental disorders [30,31,32]. The clinical features observed in affected patients in this cohort aligned with the phenotypic spectrum described for these syndromes, supporting the likely pathogenicity of these variants.

Using bioinformatics and AI-driven tools improved the interpretation of variants with uncertain significance. Consensus among traditional prediction methods, AlphaMissense scores, and protein stability analyses strengthens the evidence for the potential pathogenicity of certain missense variants. The variants GRIA3 (G730E) and CRYBA4 (L69P) were most consistently supported by these tools, while other variants showed mixed predictions. GRIA3 encodes a subunit of the AMPA-type glutamate receptor, which is crucial for excitatory neurotransmission and normal neurodevelopment. Variants affecting conserved residues in this receptor may disrupt its function, possibly contributing to the neurological features seen in some syndromic cataract cases. CRYBA4 encodes βA4-crystallin, a structural protein vital for maintaining the transparency and stability of the ocular lens. Missense variants affecting conserved residues in crystallins may interfere with interprotein interactions, leading to aggregation, instability, and lens opacity.

The potential impacts on the structural stability of the CRYBA4 p.(Leu69Pro) and GRIA3 p.(Gly730Glu) variants provide further evidence of their likely pathogenicity. In CRYBA4, replacing leucine with proline may be particularly disruptive because proline can impose rigid backbone constraints of βA4-crystallin. Since crystallins play a vital role in maintaining lens transparency, such destabilisation could increase the risk of protein degradation. In GRIA3, substituting glycine with glutamic acid introduces both an increased side-chain size and a negative charge at the affected site. This change may reduce local flexibility and interfere with normal intramolecular or intermolecular interactions necessary for receptor conformation and function. Overall, these residue-level changes support the idea that both variants may compromise protein structural integrity and contribute to disease development.

On the other hand, the inconsistent predictions for certain variants could arise from the different approaches used by bioinformatics tools, which rely on diverse features such as evolutionary conservation, structural modelling, and machine-learning datasets. Variants located in regions with moderate conservation or sparse structural information may thus produce varying predictions depending on the algorithm.

## 5. Conclusions

This study highlights the vital role of early genetic testing in children with cataracts, especially in populations with high consanguinity and elevated rates of genetic disorders. These findings support the recommendation to implement genetic testing early in paediatric cataract patients and their families, which may facilitate accurate diagnosis and prompt clinical management. Furthermore, integrating genetic screening and counselling into routine ophthalmic assessments could help identify syndromic cases and assist affected families in making informed reproductive decisions. Additional large-scale studies in regional populations are also needed to better define the genetic spectrum of paediatric cataracts and to improve future diagnostic and therapeutic strategies.

## Figures and Tables

**Table 1:** Patients Demographic and Clinical Presentation.

Patient	Age of Presentation	Unilateral/Bilateral Cataract	Congenital/Juvenile	Category
1	14 days	Bilateral	Congenital	Pure Cataract
2	34 days	Bilateral	Congenital	Ocular Cataract
3	3 weeks	Bilateral	Congenital	Ocular Cataract
4	4 years	Bilateral	Juvenile	Syndromic
5	6 years and 8 months	Bilateral	Juvenile	Ocular Cataract
6	2 years 9 months	Bilateral	Juvenile	Ocular Cataract
7	6 months	Bilateral	Congenital	Ocular Cataract
8	6 years	Bilateral	Juvenile	Ocular Cataract
9	1 month	Unilateral (left eye)	Congenital	Ocular Cataract
10	6 years	Bilateral	Juvenile	Ocular Cataract
11	6 months	Bilateral	Congenital	Ocular Cataract
12	4 months	Bilateral	Congenital	Syndromic
13	3 days	Bilateral	Congenital	Syndromic
14	2 months	Bilateral	Congenital	Syndromic
15	<1 month	Bilateral	Congenital	Syndromic
16	1 month	Bilateral	Congenital	Ocular Cataract
17	11 months	Bilateral	Congenital	Ocular Cataract
18	2 months	Bilateral	Congenital	Syndromic
19	5 days	Bilateral	Congenital	Syndromic
20	1.5 months	Bilateral	Congenital	Syndromic
21	4 years	Bilateral	Juvenile	Syndromic
22	3 months	Bilateral	Congenital	Syndromic
23	2 days	Bilateral	Congenital	Syndromic
24	At birth	Bilateral	Congenital	Syndromic
25	2 months	Bilateral	Congenital	Syndromic
26	2 months	Bilateral	Congenital	Syndromic
27	12 days	Bilateral	Congenital	Syndromic
28	14 days	Bilateral	Congenital	Syndromic

**Table 2:** Genetic Profile of Identified Cases.

Patient	Gene	Variant Change	Classification	Pattern of Inheritance	Genotype	Consanguinity	Family History
1	*CRYBB1*	c.171del (p.Asn58fs)	P	AR	Homo	Yes	No
2				AR	Homo	Yes	Yes
3				AR	Homo	Yes	No
4				AR	Homo	Yes	No
5				AR	Homo	Yes	Yes
6				AR	Homo	No	No
7				AR	Homo	Yes	No
8				AR	Homo	Yes	Yes
9				AR	Homo	No	No
0				AR	Homo	No	No
11				AR	Homo	Yes	Yes
12				AR	Homo	Yes	No
13	*SIL*	c.1030-9G>A	LP	MT	Homo	No	Yes
14	*COL18A1*	c.355delG p.(V119fs) *	P	AR	Homo	Yes	No
15		c.803C>T p.(a28v)	VUS	AR	Homo	Yes	Yes
16	*FYOC1*	c.449T>C p.(I150T)	VUS	AR	Homo	Yes	No
17	*CRYBA4*	c.206T>C p.(Leu69Pro)	VUS	AD	Hetero	No	Yes
18	*RAB3GAP2*	c.1348dup p.(Ser450Phefs*36) *	LP	AR	Homo	Yes	No
19	*PEX7*	c.694C>T p.(Arg232)	P	AD	Homo	No	No
20		c.321_322del p.(Tyro107*)	P	AR	Homo	Yes	Yes
21	*MAF*	c.188C>G p.(Pro36Arg)	VUS	AD	Hetero	No	Yes
22	*RAB3GAP1*		P	AR	Homo	Yes	Yes
23		c.1009C>T p.(Arg337*)	P	AR	Homo	Yes	No
24	*GNPAT*	c.569-3T>G g.(231401036T>G)	P	AR	Homo	Yes	No
25		c.569-3T>G g.(231401036T>G)	P	AR	Homo	Yes	Yes
26	*EPHA2*	c.987del p.(Ser330Profs*63)	LP	AD	Homo	Yes	Yes
27	*AGK*	c.424-3C>G g.(141315268C>G)	P	AR	Homo	Yes	No
28	*GRIA3*	c.2189G>C:p.(Gly730Val)	VUS	X-linked recessive	Hetero	Yes	No

Abbreviations: P: Pathogenic, LP: Likely Pathogenic, VUS: Variant of Unknown Significance, AR: Autosomal Recessive, AD: Autosomal Dominant, Homo: Homozygous, Hetero: Heterozygous, MT: Mitochondrial. * Novel Variant.

**Table 3:** Correlations Between Genotype and Phenotype in the Identified Cases.

Patient	Gene	Variant	Cataract Phenotype	Ocular Phenotype	Systemic Phenotype
1	*CRYBB1*	c.171del (p.Asn58fs)	lamellar Pulverulent	NA	NA
2			Anterior polar	Strabismus	NA
3			Anterior polar	Strabismus	NA
4			Nuclear	Glaucoma Strabismus,	Gross motor delay, dysmorphic Craniofacial features, microcephaly, developmental delay
5			Sutural	Esotropia	NA
6			Anterior polar	Glaucoma esotropia, Retinal detachment	NA
7			lamellar Pulverulent	Strabismus	NA
8			Nuclear	Glaucoma strabismus	NA
9			Nuclear	Esotropia	NA
10			Nuclear	Strabismus	NA
11			Nuclear	Esotropia	NA
12			Anterior polar	Strabismus	Musculoskeletal and cardiac abnormalities, thrombocytopenia
13	*SIL*	c.1030-9G>A	Not otherwise specified	Esotropia, optic nerve atrophy, Nystagmus,	Global developmental delay, hypotonia, ataxia, nystagmus, cerebellar atrophy
14	*COL18A1*	c.355delG p.(V119fs)	Total	Exotropia, high myopia, congenital retinal dystrophy, rotational spontaneous nystagmus,	Knobloch syndrome, speech delay, intellectual disability, craniofacial dysmorphic features, Microcephaly Hear loss
15	*COL18A1*	c.803C>T p.(a28v)	lamellar Pulverulent	Strabismus Retinal dystrophy,	Knobloch syndrome, Microcephaly Hear loss Anatomical Upper airway obstructionist
16	*FYOC1*	c.449T>C p.(I150T)	nuclear	Strabismus	NA
17	*CRYBA4*	c.206T>C p.(Leu69Pro)	Total	Strabismus, microphthalmia	NA
18	*RAB3GAP2*	c.1348dup p.(Ser450Phefs*36)	Not otherwise specified	Strabismus, optic nerve atrophy	Martsolf Syndrome, Microcephaly
19	*PEX7*	c.694C>T p.(Arg232)	Not otherwise specified	Strabismus,	Microcephaly, hypotonia, Anatomical Upper airway obstruction, musculoskeletal rhizomatic chondrodysplasia punctata type 1
20		c.321_322del p.(Tyro107*)	Anterior subcapsular	Strabismus,	Gross motor delay, dysmorphic craniofacial, Failure to thrive, Anatomical Upper airway obstruction, rhizomatic chondrodysplasia punctata type 1, hydronephrosis grade1, inguinal hernia, ASD, PDA, Developmental disability
21	*MAF*	c.188C>G p.(Pro36Arg)	Nuclear	strabismus	Ayme-Gripp syndrome, developmental delay, gross motor delay, craniofacial dysmorphic features, failure to thrive, microcephaly Musculoskeletal, Hypotonia hydrocele, depressed nasal bridge with hypertelorism, micrognathia
22	*RAB3GAP1*		Anterior Polar	Strabismus Microphthalmia	Developmental delay, Gross motor delay, Dysmorphic Craniofacial Features, Failure to thrive, brain atrophy, musculoskeletal and renal abnormalities, hypotonia, Warburg micro syndrome 1 epilepsy with hypsarrhythmia, undescended testes, developmental regression, nephrocalcinosis, severe GERD, delayed gastric emptying,
23		c.1009C>T p.(Arg337*)	Not otherwise specified	Strabismus	Gross motor delay, Failure to thrive, Microcephaly Hypotonia Developmental disability Warburg Micro syndrome type 1
24	*GNPAT*	c.569-3T>G g.(231401036T>G)	Anterior polar	Strabismus Glaucoma	developmental delay, gross motor delay, craniofacial dysmorphic features, failure to thrive, microcephaly Musculoskeletal, Hear loss Anatomical Upper airway obstruction, Rhizomatic chondrodysplasia punctata type II
25		c.569-3T>G g.(231401036T>G)	Anterior polar	Strabismus	developmental delay, gross motor delay, craniofacial dysmorphic features, failure to thrive, microcephaly Musculoskeletal, CHD Rhizomatic chondrodysplasia punctata type II
26	*EPHA2*	c.987del p.(Ser330Profs*63)	Posterior polar	Glaucoma Strabismus	Hydronephrosis
27	*AGK*	c.424-3C>G g.(141315268C>G)	Not otherwise specified	Strabismus Nystagmus	Gross motor delay, Failure to thrive, Microcephaly musculoskeletal Developmental delay Senger syndrome
28	*GRIA3*	c.2189G>C:p.(Gly730Val)	Not otherwise specifie	Strabismus	Seizures Microcephamongpotonia Creatinine deficiency ADHD IUGR Strabismus

**Table 4:** Prediction of Variant Pathogenicity of (VUS) Using Various Bioinformatics Tools.

Gene	Variant	dbSNP ID	Panther		PhD-SNP		SIFT		SNAP2		Meta-SNP	
			Preservation Time	Pred	Score	Pred	Score	Pred	Score	Pred	Score	Pred
*CRYBA4*	c.206T>C p.(Leu69Pro)	rs74315487	0.898	D	0.915	D	0.001	D	0.760	D	0.857	D
*MAF*	c.188C>G p.(Pro36Arg)	rs1057518878	0.832	D	0.571	N	0.001	D	0.821	D	0.473	N
*COL18A1*	c.803C>T p.(a23v)	rs375414087	0.100	N	0.362	N	0.150	N	0.450	N	0.094	N
*GRIA3*	c.2189G>C:p.(G730E)	rs866395967	0.907	D	0.711	D	0.001	D	0.712	D	0.714	D

**Table 5:** Alpha-missense prediction for the identified pathogenic missense variants.

Gene	dbSNP ID	Protein Change	Alpha-Missense	
			Score	Prediction
*CRYBA4*	rs74315487	L69P	0.810	Likely pathogenic
*MAF*	rs1057518878	P36R	0.333	Likely benign
*COL18A1*	rs375414087	A23V	NA	NA
*GRIA3*	rs866395967	G730E	0.991	Likely pathogenic

**Table 6:** List of variants that reduce protein stability.

Gene	dbSNP ID	Protein Changes	I-Mutant		MuPro	
			Stability	RI	Stability	Score
*CRYBA4*	rs74315487	L69P	Decrease	5	Decrease	−2.17
*GRIA3*	rs866395967	G730E	Decrease	4	Decrease	−1.103

## Data Availability

All relevant data are within the manuscript.

## Supplementary Materials

The following supporting information can be downloaded at: https://www.mdpi.com/article/10.3390/jcm15062420/s1, Table S1: Overview of Inheritance Pattern Classifications.
