# Supplementary material for: The Genetic Landscape of Paediatric Cataract in Saudi Arabia: A Two-Decade Cohort with Novel Variants, Genotype–Phenotype Correlations, and Bioinformatic Analysis
Source: J Clin Med. 2026 Mar 21;15(6):2420. doi: 10.3390/jcm15062420 (PMC13026777; doi:10.3390/jcm15062420)
Supplement: Supplementary file 1 [file jcm-15-02420-s001.zip › jcm-4183686-supplementary.pdf]

Journal of Clinical Medicine

MDPI

Supplementary Table S1:

Overview of Inheritance Pattern Classifications

Patient	Genetic Findings	Family History	Inheritance Pattern
1–12	CRYBB1, c.171del (p.Asn58fs), Homozygous	Mixed	Autosomal Recessive
13	SIL, c.1030-9G>A, Homozygous	Yes	Autosomal Recessive
14	COL18A1, c.355delG (p.Val119fs), Homozygous	No	Autosomal Recessive
15	COL18A1, c.803C>T (p.Ala28Val), Homozygous	Yes	Autosomal Recessive
16	FYCO1, c.449T>C (p.Ile150Thr), Homozygous	No	Autosomal Recessive
17	CRYBA4, c.206T>C (p.Leu69Pro), Heterozygous	Yes	Autosomal Dominant
18	RAB3GAP2, c.1348dup (p.Ser450Phefs*36), Homozygous	No	Autosomal Recessive
19	PEX7, c.694C>T (p.Arg232), Homozygous	No	Autosomal Dominant
20	PEX7, c.321_322del (p.Tyr107*), Homozygous	Yes	Autosomal Recessive
21	MAF, c.188C>G (p.Pro36Arg), Heterozygous	Yes	Autosomal Dominant
22–23	RAB3GAP1 c.1009C>T p.(Arg337*), Homozygous	Mixed	Autosomal Recessive
24–25	GNPAT, c.569-3T>G, Homozygous	Mixed	Autosomal Recessive
26	EPHA2, c.987del (p.Ser330Profs*63), Homozygous	Yes	Autosomal Dominant
27	AGK, c.424-3C>G, Homozygous	No	Autosomal Recessive
28	GRIA3, c.2189G>C (p.Gly730Val), Heterozygous	No	X-linked
